# Changes in portal pulsatility index induced by a fluid challenge in patients with haemodynamic instability and systemic venous congestion: a prospective cohort study

**DOI:** 10.1186/s13613-024-01391-2

**Published:** 2024-11-01

**Authors:** Martin Ruste, Rehane Reskot, Rémi Schweizer, Valentin Mayet, Jean-Luc Fellahi, Matthias Jacquet-Lagrèze

**Affiliations:** 1 Service d’anesthésie-réanimation, Hôpital Louis Pradel, Hospices Civils de Lyon, 59, Boulevard Pinel, 69394 Lyon Cedex, Bron France; 2 Faculté de Médecine Lyon Est, Université Claude Bernard, Lyon 1, Lyon, France; 3 Laboratoire CarMeN, Inserm UMR 1060, Université Claude Bernard, Lyon 1, Lyon, France

**Keywords:** Systemic venous congestion, Ultrasound, Haemodynamic instability, Preload responsiveness, Portal pulsatility index

## Abstract

**Background:**

It is uncertain whether fluid administration can improve patients with systemic venous congestion and haemodynamic instability. This study aimed to describe the changes in systemic venous congestion and peripheral perfusion parameters induced by a fluid challenge in these patients, and to analyse the influence of the fluid responsiveness status on these changes.

**Methods:**

The study is a single-centre prospective cohort study of 36 critically ill ICU patients with haemodynamic instability and a maximum vena cava diameter ≥ 20 mm. Changes in cardiac index during a fluid challenge (4 mL/kg of lactated Ringer’s solution during 5 min) assessed by pulse contour analysis, central venous pressure, ultrasound systemic congestion parameters (portal venous flow pulsatility index, supra hepatic and intrarenal venous Doppler), and peripheral perfusion parameters (capillary refill time and peripheral perfusion index) were assessed in the overall population. All these data were compared between patients presenting a cardiac index increase > 10% during the fluid challenge (fluid responders) and the others (fluid non-responders).

**Results:**

Twenty-eight (78%) patients were admitted for postoperative care following cardiac surgery; their mean ± SD left ventricular ejection fraction was 42 ± 9% and right ventricular dysfunction was found in at least 61% of the patients. The mean ± SD SOFA score was 9 ± 3. Thirteen (36%) patients were fluid responders. The fluid challenge administration induced a significant increase in portal pulsatility index, VExUS score, and central venous pressure without significant difference of these changes between fluid responders and non-responders. No significant change in perfusion parameters was observed.

**Conclusion:**

Fluid administration in patients with haemodynamic instability and systemic venous congestion worsens venous congestion regardless of the fluid responsiveness status, without improving perfusion parameters.

**Supplementary Information:**

The online version contains supplementary material available at 10.1186/s13613-024-01391-2.

## Background

Fluid management in critically ill patients has been described as composed of four phases (1, 2); each has specific objectives to improve the risk–benefit ratio of fluid administration or removal. During the optimisation phase, which aims to optimise tissue perfusion, the current guidelines recommend fluid administration in patients with fluid responsiveness, as this could lead to improve arterial oxygen delivery (3, 4). However, these guidelines are challenged by the emerging concept of fluid tolerance, defined as the degree to which a patient can tolerate administration of fluids without causation of organ dysfunction (5). Furthermore, fluid responsiveness can co-exist with venous congestion (6), adding to the complexity of fluid management in this context, since fluid administration may improve or worsen a same patient. Systemic venous congestion is a specific haemodynamic state associated with organ dysfunction and poor outcomes, and is probably related to several mechanisms such as intravascular hypervolemia, myocardial dysfunction, and mechanical ventilation (7–9). In addition to central venous pressure, which is the long-established marker of systemic venous congestion, ultrasound evaluation of portal, renal, and supra hepatic venous Doppler allows bedside assessment of systemic venous congestion in the ICU (10). In particular, portal venous flow pulsatility can be used as a dynamic parameter since it varies with fluid administration in healthy volunteers (11), and with end-expiratory pressure variations (12) as well as after diuretic administration in critically ill patients (13). In this context, the risk–benefit ratio of fluid administration could be assessed by simultaneously estimating cardiac index and filling pressure variations during a fluid challenge (14).

The aim of the present study was to describe the variations in systemic venous congestion parameters during a fluid challenge in patients with haemodynamic instability and systemic venous congestion. We hypothesised that the portal pulsatility index would increase more in patients without fluid responsiveness.

## Methods

### Study design

The study is a single-centre prospective cohort study of patients admitted in the ICU of the *Hôpital Louis Pradel* (Cardiovascular and thoracic centre, Hospices Civils de Lyon, Bron, France). The study protocol was registered in clinicaltrials.gov (NCT05828407) and approved by an institutional review board (Ouest-1, IRB: 0990-0279). In accordance with the French law n° 2012-300 of the 5 March 2012, the non-opposition of the patients (or their support person when the patient could not be informed) was systematically obtained before enrolment. Data management was performed in accordance with the French Data Protection Authority, Reference Method 003 (15). The reporting of the study is in accordance with the STROBE guidelines (Additional file Table 1) (16).

### Participants

To be included, patients had to fulfil all the following criteria: to present haemodynamic instability (defined as the need for vasopressor administration to maintain a mean arterial pressure > 65 mmHg and at least arterial lactate > 2 mmol/L or capillary refill time > 3 s, or mottling score > 1, or urine output < 0.5 mL/kg/h for 6 h) and to have an inferior vena cava maximum diameter > 20 mm, which defines the first degree of systemic venous congestion in VExUS score (17). The exclusion criteria were: pregnancy or breastfeeding, cirrhosis, mechanical circulatory support, severe acute pulmonary oedema (oxygen flow rate requirement > 5 L/min associated with a rapid onset of acute respiratory failure with radiological pulmonary interstitial oedema and elevated left ventricular filling pressure), inability to assess capillary refill time, severe tricuspid regurgitation, aortic dissection, legal protective measures, patient opposition to participate, and no affiliation to a health insurance or equivalent.

### Endpoints

The primary endpoint was the absolute change in portal pulsatility index (PP) during a fluid challenge, expressed as a percentage and defined as PP = 100 × (Vmax − Vmin/Vmax), where Vmax is the peak velocity and Vmin is the nadir velocity in one cardiac cycle. Secondary endpoints were the variations observed in other systemic venous congestion parameters (supra hepatic systolic to diastolic wave ratio, intrarenal venous Doppler, VExUS score) as well as in peripheral perfusion parameters (capillary refill time and peripheral perfusion index). The effect of the fluid responsiveness status on variations in systemic venous congestion and perfusion parameters during the fluid challenge were also analysed.

### Protocol and data collection

The following variables were collected at baseline: anthropometric characteristics, admission category, Sepsis Organ Failure Assessment (SOFA) score (18), cumulative fluid balance defined as the weight variation between the weight before the admission (or at the admission) and the weight at inclusion, arterial lactate, veno-arterial PCO_2_ gap and organ support, and echocardiographic data. Before the fluid challenge, haemodynamic data (cardiac and stroke volume index form pulse contour analysis, invasive arterial pressure, central venous pressure, and heart rate), ultrasound venous congestion parameters as well as perfusion parameters (capillary refill and peripheral perfusion index) were collected. The fluid challenge consisted in the administration of 4 mL/kg of balanced crystalloid (lactated Ringer’s solution at room temperature) during five minutes with the re-evaluation of the haemodynamic data in the first minute after the administration, and of the other parameters in the following minutes, starting with PP. Fluid responsiveness was defined as a > 10% increase in cardiac index during fluid challenge (14), estimated by pulse contour analysis using FloTrac™ transducer / Hemosphere™ monitor (Edwards Lifesciences, Irvine, CA, USA) or PiCCO^®^ transducer and monitor (Pulsion Medical Systems, Munich, Germany). Capillary refill time was estimated on the fingertip using a chronometer, by applying a firm pressure, blanching the nail of the operator, with at least two measurements averaged (19), and peripheral perfusion index (20) was estimated using photoplethysmography derived from Intellivue MX750 monitor (Philips Healthcare, Andover, MA, USA).

### Ultrasound measurements

Ultrasound measurements were carried out using Vivid™ S6 (GE Healthcare, Madison, WI, USA), by 4 of the co-authors (RR, VM, MR, MJL), each with advanced or expert competence in critical care echocardiography (21). All measurements were obtained during end-expiration in the semi-recumbent position (30°), and for echocardiography, with concomitant electrocardiographic recording. Transthoracic echocardiography was performed at baseline to assess left ventricular systolic function (visual estimation of left ventricular ejection fraction), left ventricular filling pressure (mitral E and A wave velocities, lateral and medial e’ velocity), right ventricular systolic function (systolic tricuspid S’ velocity, tricuspid annular plane systolic excursion, right ventricular fractional area change, tricuspid regurgitation velocity), and cardiac output (left ventricular outflow tract velocity time integral). Regarding ultrasound measurements of the systemic venous congestion, the diameter of the inferior vena cava was measured at its intrahepatic portion, 2 cm from the junction with the right atrium, in a longitudinal view, using a subxiphoid or transhepatic view. The assessment of portal venous flow, supra hepatic venous flow, and intrarenal venous flow were performed between the 9th and 11th intercostal spaces on the right axillary line, as previously described (22). Such an approach allows to describe the PP as a quantitative variable, the supra hepatic systolic to diastolic wave ratio as a quantitative variable, the pattern of intrarenal venous Doppler as an ordinal categorical variable (type 1: continuous flow pattern; type 2: biphasic flow pattern; type 3: monophasic diastolic pattern), and the VExUS score as an ordinal categorical variable between 0 and 3 reflecting the degree of systemic venous congestion (17).

### Statistical analysis

The data distribution was estimated using skewness and kurtosis analysis, and the Shapiro–Wilk test. Data are expressed as mean ± standard deviation (SD), median [25th to 75th percentile], or count and percentage, as appropriate. Missing values were taken into account without imputation. The sample size was calculated to compare the variation in PP in fluid responders and fluid non-responders. We estimated that a 20 (± 15) % increase in PP was clinically significant (12), defining an effect size of 1.2. For an alpha risk of 0.05 and a beta risk of 0.9, we estimated that 12 patients would be required in each group. Anticipating a frequency of fluid responsiveness in our population of 33%, we estimated a sample size of 36 patients. Changes during the fluid challenge were analysed using the Wilcoxon signed-rank test and presented with paired boxplots showing individual trajectories between before and after the fluid challenge. Differences between fluid responders and non-responders, as defined above, were analysed by comparing the absolute changes during the fluid challenge between the two groups using the Mann–Whitney U test. A sensitivity analysis was carried out by defining fluid responsiveness as an increase in stroke volume index > 10% during the fluid challenge. Relationships between absolute changes during the fluid challenge in haemodynamic, systemic venous congestion, and perfusion parameters were assessed using the Spearman correlation coefficient and presented in a correlation matrix. The statistical analysis was performed using R version 4.3.2 (R Core Team, 2017, Vienna, Austria). All the tests were two-sided and a *P*-value < 0.05 was considered significant.

## Results

**Fig. 1:**
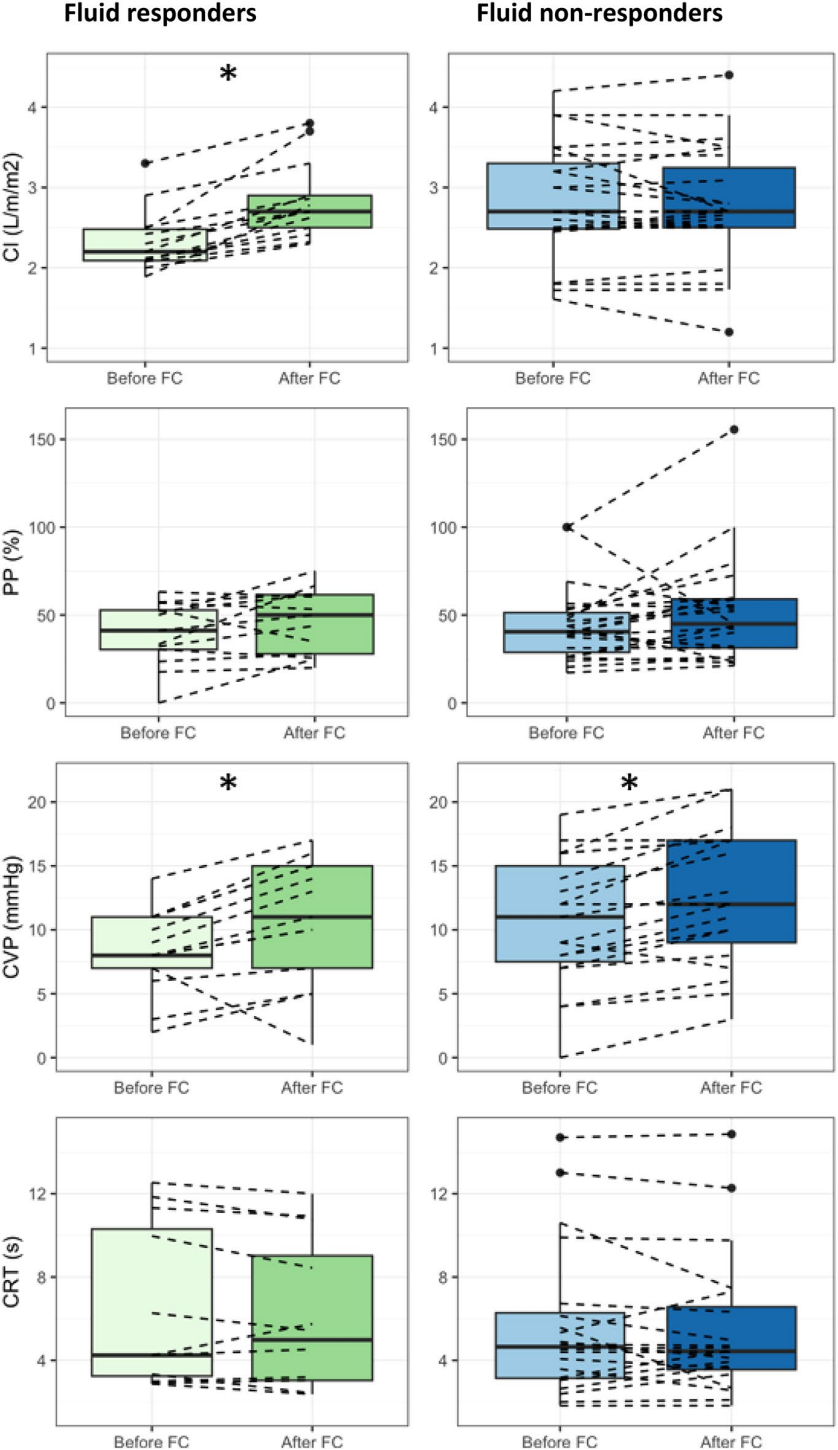
Variations of the cardiac index, portal pulsatility index, central venous pressure, and capillary refill time parameters during the fluid challenge. Left column: fluid responders. Right column: fluid non-responders. CI: cardiac index; CRT: capillary refill time; CVP: central venous pressure; FC: fluid challenge; PP: portal pulsatility index; *P value < 0.05 for Wilcoxon signed-ranked test for the comparison between before and after fluid challenge

**Fig. 2:**
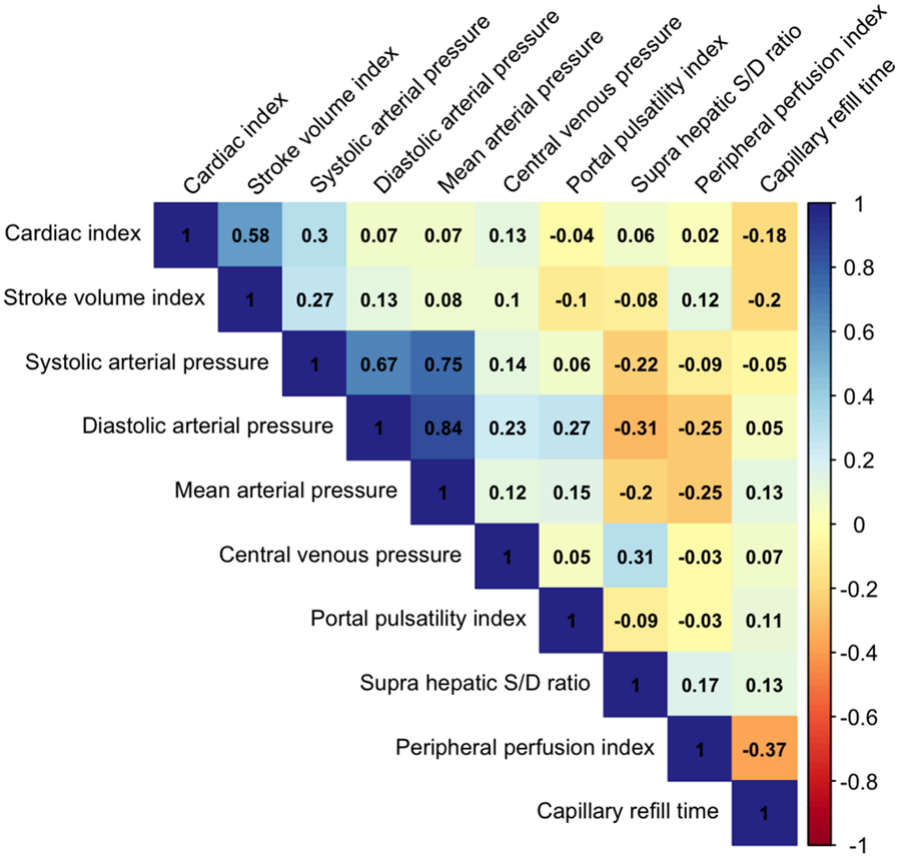
Correlation matrix to explore the relationship between the changes in haemodynamic, perfusion, and ultrasound estimated systemic venous congestion parameters during the fluid challenge. Numerical values correspond to Spearman’s rank correlation coefficient

Thirty-six patients were enrolled between 10 July 2023 and 03 June 2024. Missing values are reported in Additional files (Table 2 and Fig. 1). Due to poor echogenicity, there was a high rate of missing values for echocardiographic variables. Therefore, a right ventricular dysfunction was defined as the presence of at least one of the following parameters: right ventricular fractional area change < 35%, tricuspid annular plane exclusion < 16 mm, or tricuspid annular systolic excursion velocity < 10 cm/s (23). A total of 72% of the patients were male and 78% were admitted to the ICU for postoperative care after a cardiac surgery. The mean ± SD left ventricular ejection fraction was 42 ± 9% and a right ventricular dysfunction was found in at least 61% of the patients. The mean ± SD SOFA score was 9 ± 3 and the median [25th–75th percentile] arterial lactate was 2.6 [2.1 to 3.2] mmol/L. Thirteen (36%) patients had a cardiac index increase > 10% after the fluid challenge and were considered as fluid responders (Table 1). Median central venous pressure, systolic as well as mean arterial pressure increased in the overall population, without differences between fluid responders and non-responders (Table 2 and Fig. 1). Furthermore, in the overall cohort, the fluid challenge administration induced a significant increase in PP (median PP [25th–75th percentile] before fluid challenge 41 [29, 53] % and 48 [30, 60] % after, P value 0.01; Table 3 and Fig. 1) and in VExUS score (P value 0.02; Table 3). Of note, there was no significant change in PP within fluid responders (P value 0.09) and within fluid non-responders (P value 0.08). In addition, between fluid responders and non-responders no difference regarding change in PP was observed (P value 0.91; Table 3 and Fig. 1). No significant change was observed in perfusion parameters (Table 2). The results of the sensibility analysis considering the fluid responsiveness status based on the stroke volume index variation are reported in Additional files Table 3. Exploratory analyses of the relationship between changes in cardiac index, venous congestion, and perfusion parameters during fluid challenge are shown in Fig. 2.

**Table 1:** Population characteristics at baseline

Variable	All (n = 36)	Fluid responders (n = 13)	Fluid non-responders (n = 23)	P value*
Age, years	69 [63, 77]	62 [60, 76]	70 [65, 77]	0.17
Height, cm	172 ± 9	176 [171, 180]	173 [168, 175]	0.25
Weight, kg	72 [60, 85]	73 [62, 82]	70 [59, 86]	0.92
Sex, female, n (%)	10 (28)	4 (31)	6 (26)	1
Body Mass Index, kg/m^2^	25 ± 4	23 [21, 27]	25 [21, 28]	0.66
Admission category, n (%)				0.39
Cardiac surgery	28 (78)	12 (92)	16 (70)	
Other surgery	1 (3)	0 (0)	1 (4)	
Medical	7 (19)	1 (8)	6 (26)	
SOFA score	9 ± 3	8 [6, 11]	9 [6, 11]	0.90
Cumulative fluid balance, body weight % **	0 [0, 8]	7 [0, 10]	0 [0, 7]	0.30
Left ventricular ejection fraction, %	42 ± 9	40 [40, 45]	45 [37, 50]	0.69
Right ventricular dysfunction, n (%) ***				0.39
Yes	22 (61)	8 (62)	14 (61)	
No	4 (11)	0 (0)	4 (17)	
Missing	10 (28)	5 (38)	5 (22)	
Arterial lactate, mmol/L	2.6 [2.1, 3.2]	2.9 [2.6, 3.6]	2.5 [1.7, 2.8]	0.046
Veno-arterial PCO_2_ gap, mmHg	7 [5, 9]	8 [5, 10]	7 [5, 8]	0.37
Norepinephrine equivalent, µg/kg/min ****	0.20 [0.09, 0.52]	0.26 [0.06, 0.48]	0.20 [0.09, 0.52]	0.97
Dobutamine, µg/kg/min	4 [0, 6]	5 [0, 8]	3 [0, 5]	0.63
Vasoactive inotropic score*****	21 [10, 60]	28 [10, 58]	20 [12, 52]	0.92
Mechanical ventilation, yes, n (%)	21 (58)	10 (77)	11 (48)	0.18
End-expiratory pressure, cmH_2_O	5 [0, 5]	5 [5]	5 [0, 5]	0.13
Renal replacement therapy, yes, n (%)	5 (14)	1 (8)	4 (17)	0.76
Inferior vena cava diameter (maximum), mm	23 [22, 25]	23.00 [22, 25]	23 [22, 24]	0.50

Values are expressed as median [25th–75th percentile], mean ± standard deviation, or count (%). * for the comparison between fluid responders and non-responders; SOFA: Sepsis Organ Failure Assessment (18); ** Estimated with the weight variation between the reference and the weight at the inclusion; *** See text for definition; **** As described by Kotani et al. (40); ***** As described by Koponen et al. (41)

**Table 2:** Haemodynamic variations observed during the fluid challenge

Variable	All (n = 36)		Fluid responders (n = 13)		Fluid non-responders (n = 23)	
	Before	After	Before	After	Before	After
Median cardiac index, L/min/m^2^ [25th to 75th percentile]	2.50 [2.12, 3.05]	2.70 [2.5, 3.14]^a^	2.20 [2.09, 2.48]	2.70 [2.50, 2.90]^a^	2.70 [2.49, 3.30]	2.70 [2.50, 3.24]^b^
Median stroke volume index, mL/m^2^ [25th to 75th percentile]	28 [25, 41]	31 [26, 41]^a^	27 [21, 37]	30 [23, 41]^a^	29 [26, 46]	31 [26, 44]^a,b^
Median heart rate, /min [25th to 75th percentile]	93 [83, 105]	91 [81, 103]^a^	91 [90, 107]	91 [90, 104]	95 [80, 102]	94 [78, 103]
Median systolic arterial pressure, mmHg [25th to 75th percentile]	103 [99, 112]	111 [103, 121]^a^	104 [99, 109]	111 [106, 120]^a^	103 [98, 117]	111 [101, 121]
Median diastolic arterial pressure, mmHg [25th to 75th percentile]	53 [47, 58]	54.5 [49, 60]^a^	52 [50, 56]	57 [53, 60]^a^	54 [46, 59]	53 [48, 59]
Median mean arterial pressure, mmHg [25th to 75th percentile]	67 [63, 72]	71 [68, 76]^a^	67 [66, 71]	72 [70, 75]^a^	68 [62, 73]	71 [66, 76]^a^
Median central venous pressure, mmHg [25th to 75th percentile]	9 [7, 12]	12 [8, 16]^a^	8 [7, 11]	11 [7, 15]^a^	11 [7, 15]	12 [9, 17]^a^
Median capillary refill time, sec [25th to 75th percentile]	4.5 [3.1, 7.5]	4.5 [3.2, 7.3]	4.2 [3.2, 10.3]	5 [3, 9]	4.6 [3.1, 6.3]	4.4 [3.6, 6.6]
Median peripheral perfusion index, % [25th to 75th percentile]	0.97 [0.33, 1.67]	1.05 [0.42, 1.50]	1.33 [0.26, 2.18]	1.30 [0.36, 1.92]	0.90 [0.56, 1.40]	[0.60, 1.30]

^a^P value < 0.05 for Wilcoxon signed-ranked test for the comparison between before and after fluid challenge

^b^P value < 0.05 for Wilcoxon rank sum test for the comparison of the absolute variation during fluid challenge between fluid responders and non-responders

**Table 3:** Variations observed in abdominal venous ultrasound measurements during the fluid challenge

Variable	All (n = 36)		Fluid responders (n = 13)		Fluid non-responders (n = 23)	
	Before	After	Before	After	Before	After
Median portal pulsatility index, % [25th to 75th percentile]	41 [29, 53]	48 [30, 60]^a^	41 [30, 53]	50 [28, 62]	40 [29, 51]	45 [31, 59]
Median S/D supra hepatic wave ratio, [25th to 75th percentile]	− 0.72 [− 0.96, 0.74]	− 0.57 [− 0.85, 0.39]	− 0.87 [− 1.03, 0.22]	− 0.76 [− 0.87, − 0.24]	− 0.69 [− 0.85, 0.74]	− 0.54 [− 0.78, 0.54]
VExUS score, n (%)						
1	8 (22)	5 (14)	2 (15)	1 (8)	6 (26)	4 (17)
2	13 (36)	12 (33)	6 (46)	6 (46)	7 (30)	6 (26)
3	15 (42)	19 (53)^a^	5 (38)	6 (46)	10 (43)	13 (56)
Intrarenal venous pattern, n (%)	All (n = 33)		Fluid responders (n = 12)		Fluid non-responders (n = 18)	
1	5 (15)	4 (12)	4 (33)	3 (25)	1 (5)	1 (5)
2	16 (48)	17 (51)	5 (42)	7 (58)	11 (52)	10 (48)
3	12 (36)	12 (36)	3 (25)	2 (17)	9 (43)	10 (48)

^a^P value < 0.05 for Wilcoxon signed-ranked test for the comparison between before and after fluid challenge

^b^P value < 0.05 for Wilcoxon rank sum test for the comparison of the absolute variation during fluid challenge between fluid responders and non-responders

NA: not applicable

## Discussion

The present study reported that in patients with systemic venous congestion and haemodynamic instability, a 4 mL/kg infusion of lactated Ringer’s solution led to a slight but significant increase in PP and central venous pressure, independently of the fluid responsiveness status; perfusion parameters did not improve, even though cardiac index and/or systemic arterial pressure increased.

These results confirm the challenge of haemodynamic management in these patients. In patients with hypoperfusion due to cardiovascular insufficiency, increasing the oxygen delivery is a cornerstone of fluid management. The first line therapy is fluid administration to increase cardiac output, since it is the main modifiable determinant of oxygen delivery. However, fluid administration can lead to interstitial fluid accumulation (24) as well as hypervolemia and/or venous congestion, all responsible for worsening tissue perfusion (7, 25). Currently, it is well established that fluid administration should be guided by dynamic parameters to reserve it for fluid responders (3, 4, 26). However, there is a lack of consensus regarding when to withdraw fluid administration in these patients. More than 40 years ago, in a central venous pressure-guided algorithm, experts proposed the “5-2” rules, suggesting to stop fluid administration when the central venous pressure increased by more than 5 cmH_2_O during a fluid challenge (27). This approach was then adapted to cardiac output-guided strategy, without a well-defined cut-off in venous congestion parameters to stop the fluid administration (14). Central venous pressure does not predict fluid responsiveness, particularly in the range observed in the present population (28, 29). Moreover, increases in central venous pressure do not predict increases in cardiac output during a passive leg raising test (30). It is therefore not surprising that during a fluid challenge the changes in the ultrasound parameters of systemic venous congestion did not differ between fluid responders and non-responders, as they have been correlated with right atrial pressure (31). Since it is unlikely that the intrinsic myocardial performance presented significant changes during the fluid challenge, the observed increase in systemic venous congestion parameters was probably related to the fluid-induced change in intravascular blood volume. The cardiac index decreased in a very small proportion of the present population, which does not support that the fluid challenge is responsible for a volume-induced right ventricular failure. Therefore, the increase in systemic venous congestion parameters is probably induced by an increase in stressed volume, with (at least in patients with preserved or increased cardiac output) an increase in the mean systemic filling pressure, as supported by the Guyton’s model (32). This could explain that both central venous pressure and mean systemic filling pressure have been associated with organ dysfunction, especially in critically ill patients with acute kidney injury (33, 34). We hypothesised that fluid responders would have a smaller increase in systemic venous congestion, based on the Levy’s approach, which suggests that cardiac output is the primary determinant of right atrial pressure (35). A previous study in healthy volunteers has reported, after fluid administration, an increase in PP only in fluid non-responders as well as an increase in portal flow only in fluid responders (11). However, the results of the present study do not support this assumption. The changes in PP during the fluid challenge were lower than 20%, the threshold that was considered herein as clinically relevant in the sample size estimation. This could explain why PP did not significantly increase within the fluid responders and non-responders groups, the study being underpowered. However, the change in PP observed was in the range of the changes observed 2 h after a successful diuretic administration (13) or induced by an increase in end-expiratory pressure from 0 to 5 cmH_2_O (12).

Herein, in the overall population and more interestingly in fluid responders, there was no significant variation in the perfusion parameters estimated during the fluid challenge. While a fluid-induced increase in oxygen delivery is not always associated with an increase in oxygen consumption (36) and microvascular perfusion (37), these results do not support a fluid administration in patients with haemodynamic instability and systemic venous congestion. It is noteworthy that peripheral perfusion index as well as capillary refill time have not been well studied in patients with systemic venous congestion. Furthermore, peripheral perfusion index theoretically reflects pulsatile to non-pulsatile blood vessels in the fingertip, which could have been modified, in opposite directions, by an increase in cardiac output and in systemic venous congestion (20).

The present study has several limitations. First, it is a single-centre study of a highly selected population with a high proportion of patients with cardiac dysfunction, which could limit the generalisability of the results. However, this was expected, since the eligibility criteria favoured such a patient profile (9, 38). Second, the eligibility criteria, and in particular the definition of systemic venous congestion, based on the maximum vena cava diameter, could be criticised. It was chosen since it has been described as the first stage of systemic venous congestion in the VExUS score (17), but collapsibility or other thresholds for diameter could be found in the literature (39). Third, it must be more considered as a mechanistically-focused study, as the sample size and the design did not allow for analysis of outcomes. Nevertheless, the study provided insights in a population with a poor prognosis (6). It showed the lack of consistency in the variations of systemic venous congestion parameters, cardiac output, and perfusion parameters during fluid administration. In this context, the main goal to be achieved to optimise haemodynamic status remains to be determined.

## Conclusion

In patients with haemodynamic instability and systemic venous congestion, a fluid challenge worsens systemic venous congestion in fluid responder and non-responder patients without improving perfusion.

## Supplementary Information

Additional file 1.

## Data Availability

All de-identified datasets may be available for secondary analysis upon reasonable request to the corresponding author.

## Abbreviations

ICU: Intensive care unit
PP: Portal pulsatility index
SOFA: Sepsis organ failure assessment
VExUS: Venous excess ultrasound grading system
