# Supplementary material for: Changes in portal pulsatility index induced by a fluid challenge in patients with haemodynamic instability and systemic venous congestion: a prospective cohort study
Source: Ann Intensive Care. 2024 Nov 1;14:167. doi: 10.1186/s13613-024-01391-2 (PMC11530414; doi:10.1186/s13613-024-01391-2)
Supplement: Supplementary file 1 — Additional file 1. [file 13613_2024_1391_MOESM1_ESM.docx]

**Additional files**

Table 1: STROBE statement checklist

|  | | Item No | Recommendation | Page No |
| --- | --- | --- | --- | --- |
| **Title and abstract** | | 1 | (*a*) Indicate the study’s design with a commonly used term in the title or the abstract | 1 |
|  |  |  | (*b*) Provide in the abstract an informative and balanced summary of what was done and what was found |  |
| Introduction | | | | |
| Background/rationale | | 2 | Explain the scientific background and rationale for the investigation being reported | 2 |
| Objectives | | 3 | State specific objectives, including any prespecified hypotheses | 3 |
| Methods | | | | |
| Study design | | 4 | Present key elements of study design early in the paper | 4 |
| Setting | | 5 | Describe the setting, locations, and relevant dates, including periods of recruitment, exposure, follow-up, and data collection | 4 |
| Participants | | 6 | (*a*) Give the eligibility criteria, and the sources and methods of selection of participants. Describe methods of follow-up | 4 |
|  |  |  | (*b*) For matched studies, give matching criteria and number of exposed and unexposed | NA |
| Variables | | 7 | Clearly define all outcomes, exposures, predictors, potential confounders, and effect modifiers. Give diagnostic criteria, if applicable | 5 |
| Data sources/ measurement | | 8* | For each variable of interest, give sources of data and details of methods of assessment (measurement). Describe comparability of assessment methods if there is more than one group | 5-6 |
| Bias | | 9 | Describe any efforts to address potential sources of bias | 7 |
| Study size | | 10 | Explain how the study size was arrived at | 7 |
| Quantitative variables | | 11 | Explain how quantitative variables were handled in the analyses. If applicable, describe which groupings were chosen and why | 7 |
| Statistical methods | | 12 | (*a*) Describe all statistical methods, including those used to control for confounding | 7 |
|  |  |  | (*b*) Describe any methods used to examine subgroups and interactions | NA |
|  |  |  | (*c*) Explain how missing data were addressed | 7 |
|  |  |  | (*d*) If applicable, explain how loss to follow-up was addressed | NA |
|  |  |  | (*e*) Describe any sensitivity analyses | 7 |
| Results | | | |  |
| Participants | | 13* | (a) Report numbers of individuals at each stage of study—eg numbers potentially eligible, examined for eligibility, confirmed eligible, included in the study, completing follow-up, and analysed | 8 |
|  |  |  | (b) Give reasons for non-participation at each stage | NA |
|  |  |  | (c) Consider use of a flow diagram | ** |
| Descriptive data | | 14* | (a) Give characteristics of study participants (eg demographic, clinical, social) and information on exposures and potential confounders | 7 |
|  |  |  | (b) Indicate number of participants with missing data for each variable of interest | 28-29 |
|  |  |  | (c) Summarise follow-up time (eg, average and total amount) | NA |
| Outcome data | | 15* | Report numbers of outcome events or summary measures over time | 8 |
| Main results | 16 | (*a*) Give unadjusted estimates and, if applicable, confounder-adjusted estimates and their precision (eg, 95% confidence interval). Make clear which confounders were adjusted for and why they were included | | NA |
|  |  | (*b*) Report category boundaries when continuous variables were categorized | | NA |
|  |  | (*c*) If relevant, consider translating estimates of relative risk into absolute risk for a meaningful time period | | NA |
| Other analyses | 17 | Report other analyses done—eg analyses of subgroups and interactions, and sensitivity analyses | | 31-32 |
| Discussion | | | | |
| Key results | 18 | Summarise key results with reference to study objectives | | 9-10 |
| Limitations | 19 | Discuss limitations of the study, taking into account sources of potential bias or imprecision. Discuss both direction and magnitude of any potential bias | | 11 |
| Interpretation | 20 | Give a cautious overall interpretation of results considering objectives, limitations, multiplicity of analyses, results from similar studies, and other relevant evidence | | 9-10-11 |
| Generalisability | 21 | Discuss the generalisability (external validity) of the study results | | 11 |
| Other information | | | | |
| Funding | 22 | Give the source of funding and the role of the funders for the present study and, if applicable, for the original study on which the present article is based | | 14 |

**Give information separately for exposed and unexposed groups.*

******** *Flow diagram is not available, due to the very transient nature of the eligibility criteria and the availability of investigators.*

*NA: not applicable.*

Table 2: missing values (except for transthoracic echocardiography measurements)

| Variables | n (%) |
| --- | --- |
| Age | 0 (0) |
| Sex | 0 (0) |
| Weight | 0 (0) |
| Admission category | 0 (0) |
| SvO_2_ | 2 (6) |
| Arterial lactate | 0 (0) |
| pCO_2_ gap | 2 (6) |
| Systolic arterial pressure before fluid challenge | 0 (0) |
| Diastolic arterial pressure before fluid challenge | 0 (0) |
| Mean arterial pressure before fluid challenge | 0 (0) |
| Central venous pressure before fluid challenge | 4 (11) |
| Heart rate before fluid challenge | 0 (0) |
| Capillary refill time before fluid challenge | 4 (11) |
| Cardiac index before fluid challenge | 0 (0) |
| Stroke volume before fluid challenge | 0 (0) |
| Peripheral perfusion pressure before fluid challenge | 2 (6) |
| Norepinephrine administration before fluid challenge | 0 (0) |
| Dobutamine administration before fluid challenge | 0 (0) |
| End expiratory pressure before fluid challenge | 0 (0) |
| Inferior vena cava diameter (max) before fluid challenge | 0 (0) |
| Inferior vena cava diameter (min) before fluid challenge | 1 (3) |
| Portal pulsatility index before fluid challenge | 0 (0) |
| Supra hepatic systolic wave velocity before fluid challenge | 2 (6) |
| Supra hepatic diastolic wave velocity before fluid challenge | 2 (6) |
| Intra renal venous systolic velocity before fluid challenge | 4 (11) |
| Intra renal venous diastolic velocity before fluid challenge | 4 (11) |
| Intra renal venous flow pattern before fluid challenge | 3 (8) |
| Systolic arterial pressure after fluid challenge | 0 (0) |
| Diastolic arterial pressure after fluid challenge | 0 (0) |
| Mean arterial pressure after fluid challenge | 0 (0) |
| Central venous pressure after fluid challenge | 4 (11) |
| Heart rate after fluid challenge | 0 (0) |
| Capillary refill time after fluid challenge | 4 (11) |
| Cardiac index before after fluid challenge | 0 (0) |
| Stroke volume before after fluid challenge | 0 (0) |
| Peripheral perfusion pressure after fluid challenge | 2 (6) |
| Inferior vena cava diameter (max) after fluid challenge | 0 (0) |
| Inferior vena cava diameter (min) after fluid challenge | 1 (3) |
| Portal pulsatility index after fluid challenge | 0 (0) |
| Supra hepatic systolic wave velocity after fluid challenge | 2 (6) |
| Supra hepatic diastolic wave velocity after fluid challenge | 2 (6) |
| Intra renal venous systolic velocity after fluid challenge | 4 (11) |
| Intra renal venous diastolic velocity after fluid challenge | 4 (11) |
| Intra renal venous flow pattern after fluid challenge | 3 (8) |

Figure 1: Missing values for transthoracic echocardiography measurements


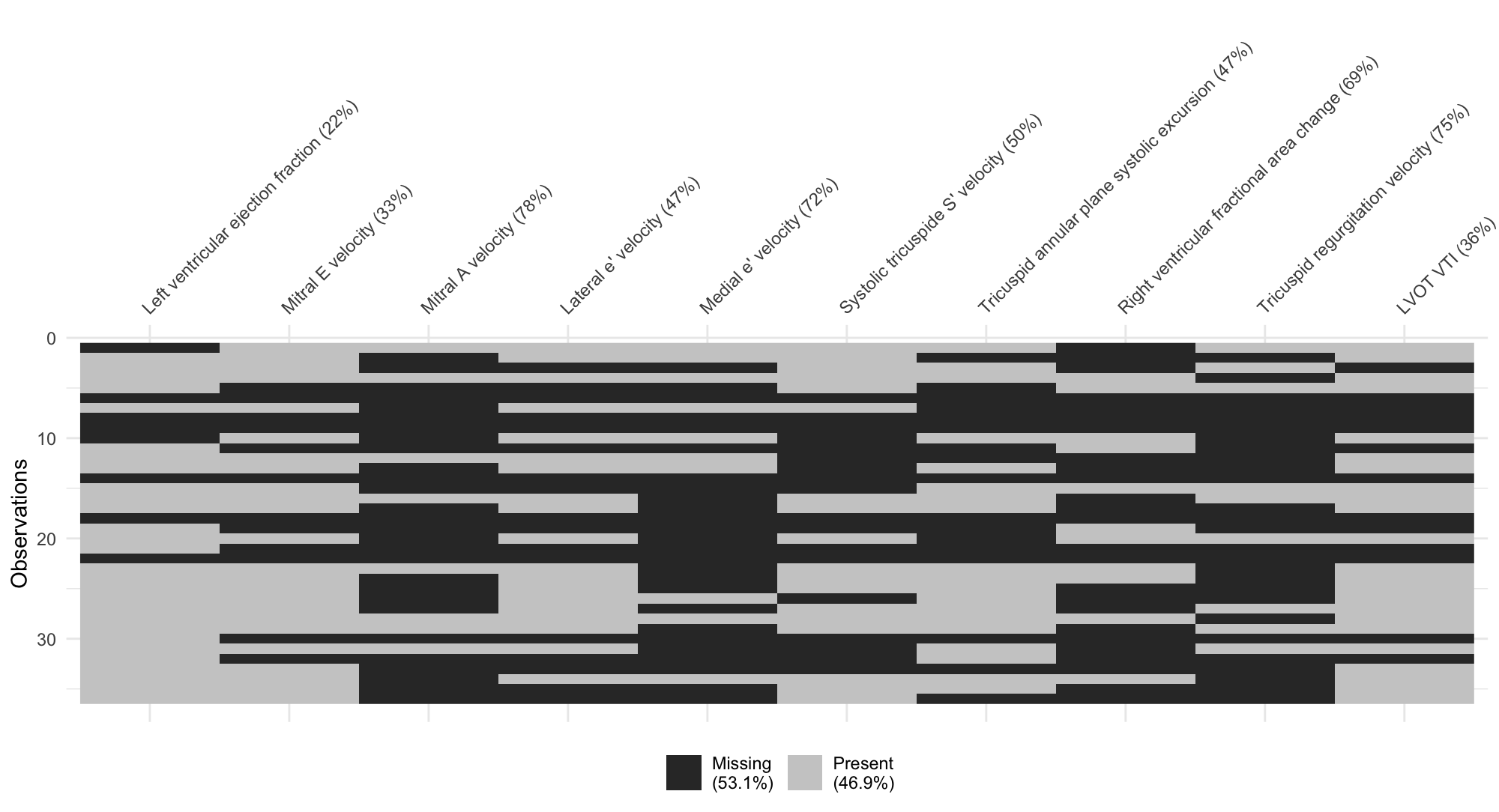


*Percentage of missing values are provided between brackets. LVOT VTI: left ventricular outflow tract velocity time integral.*

Table 3: Changes in systemic venous congestion and perfusion parameters observed during the fluid challenge for a definition of fluid responsiveness based on stroke volume index variations.

| Variable | Baseline | After the fluid challenge | P value **^a^** | P value **^b^** |
| --- | --- | --- | --- | --- |
| Median stroke volume index, mL/m^2^ [25^th^ to 75^th^ percentile] |  |  |  | <0.01 |
| Fluid responders (n=17) | 28 [25, 37] | 31 [30, 41] | <0.01 |  |
| Fluid non-responders (n=19) | 29 [25, 46] | 31 [26, 44] | 0.18 |  |
| Median portal pulsatility index, % [25^th^ to 75^th^ percentile] |  |  |  | 0.86 |
| Fluid responders (n=17) | 40 [26, 52] | 44 [26, 60] | 0.047 |  |
| Fluid non-responders (n=19) | 44 [34, 54] | 52 [32, 59] | 0.12 |  |
| Median central venous pressure, mmHg [25^th^ to 75^th^] |  |  |  |  |
| Fluid responders (n=15) | 8 [5, 11] | 10 [6, 15] | <0.01 |  |
| Fluid non-responders (n=16) | 11 [8, 14] | 13 [10, 17] | <0.01 |  |
| Median S/D supra hepatic wave ratio, [25^th^ to 75^th^] |  |  |  | 0.70 |
| Fluid responders (n=16) | - 0.81 [-0.96, 0.89] | - 0.65 [-0.86, -0.24] | 0.71 |  |
| Fluid non-responders (n=18) | -0.69 [-0.93, 0.61] | -0.51 [-0.83, 0.71] | 0.32 |  |
| Intra renal venous pattern (count, %) |  |  |  | NA |
| Fluid responders (n=16) |  |  | 0.77 |  |
| 1 | 4 (25) | 2 (12) |  |  |
| 2 | 7 (44) | 10 (62) |  |  |
| 3 | 5 (31) | 4 (25) |  |  |
| Fluid non-responders (n=17) |  |  | 1 |  |
| 1 | 1 (6) | 2 (12) |  |  |
| 2 | 9 (53) | 7 (41) |  |  |
| 3 | 7 (41) | 8 (47) |  |  |
| VExUS score (count, %) |  |  |  | NA |
| Fluid responders (n=17) |  |  | 0.07 |  |
| 1 | 3 (18) | 1 (6) |  |  |
| 2 | 8 (47) | 8 (47) |  |  |
| 3 | 6 (35) | 8 (47) |  |  |
| Fluid non-responders (n=19) |  |  | 0.23 |  |
| 1 | 5 (26) | 4 (21) |  |  |
| 2 | 5 (26) | 4 (21) |  |  |
| 3 | 9 (47) | 11 (58) |  |  |
| Median capillary refill time, sec [25^th^ to 75^th^ percentile] |  |  |  | 0.75 |
| Fluid responders (n=16) | 4.2 [3.2, 10.3] | 4.6 [3, 9] | 0.29 |  |
| Fluid non-responders (n=16) | 4.7 [3.1, 4.8] | 4.4 [3.6, 6.6] | 0.62 |  |
| Median peripheral perfusion index, % [25^th^ to 75^th^ percentile] |  |  |  | 0.85 |
| Fluid responders (n=16) | 1.04 [0.31, 1.95] | 1.15 [0.36, 1.75] | 0.89 |  |
| Fluid non-responders (n=18) | 0.90 [0.56, 1.55] | 0.90 [0.60, 1.37] | 0.85 |  |

***^a^*** *Wilcoxon signed-ranked test for the comparison between before and after fluid challenge;* ***^b^*** *Wilcoxon rank sum test for the comparison of the absolute variation during fluid challenge between fluid responders and non-responders, as defined by a stroke volume index increase > 10% during the fluid challenge.*
